# Retraction: Transcriptome analysis reveals a role for the endothelial ANP-GC-A signaling in interfering with pre-metastatic niche formation by solid cancers

**DOI:** 10.18632/oncotarget.28223

**Published:** 2022-03-24

**Authors:** Takashi Nojiri, Miki Arai, Yutaka Suzuki, Motofumi Kumazoe, Takeshi Tokudome, Koichi Miura, Jun Hino, Hiroshi Hosoda, Mikiya Miyazato, Meinoshin Okumura, Shinpei Kawaoka, Kenji Kangawa

**Affiliations:** ^1^Department of Biochemistry, National Cerebral and Cardiovascular Center Research Institute, Suita-City, Osaka, Japan; ^2^Department of General Thoracic Surgery, Osaka University Graduate School of Medicine, Suita-City, Osaka, Japan; ^3^Advanced Telecommunications Research Institute International (ATR), The Thomas N. Sato BioMEC-X Laboratories, Kyoto, Japan; ^4^The University of Tokyo, Graduate School of Frontier Science, Kashiwa, Japan; ^5^Department of Regenerative Medicine and Tissue Engineering, National Cerebral and Cardiovascular Center Research Institute, Suita-City, Osaka, Japan; ^6^ERATO Sato Live Bio-Forecasting Project, Japan Science and Technology Agency, Kyoto, Japan; ^*^These authors contributed equally to this work and are co-first authors

**This article has been retracted**: The National University Corporation Osaka University and National Cerebral and Cardiovascular Center Hospital in Osaka, Japan, investigated the work of the researcher Takashi Nojiri. Several of his articles in other journals have been retracted/corrected. One article of Takashi Nojiri was published in Oncotarget. https://doi.org/10.18632/oncotarget.18032. Based on the investigation results, which concluded there were several instances of intentional data falsification, Oncotarget has decided to retract this article. The NCVC committee notes, “We believe this paper should be withdrawn, and we have communicated our stance to the lead and corresponding authors.”

Specific concerns of the committee are noted below:

1. Issues (data forgery, falsification) related to Supplementary Figure 4B and Figure 7C: Data from another group were partially used to create secondary data, and discrepancies were discovered between the original data and the data included in the initially submitted version of the manuscript. Furthermore, it is unlikely that the act of acquiring partial data from a treatment group and using it as data representing another group was done unintentionally; therefore, in this case, data falsification was recognized as the result of a deliberate act.

2. Issues (data forgery, falsification) related to Supplementary Figures 9B and 13B: This section revealed a discrepancy between the original data and those used in the initially submitted version of the manuscript. Additionally, all four items, which were believed to exhibit statistically significant differences, actually exhibited no significant difference at allowing to the discrepancies. This inconsistency could not possibly have been caused by a simple misstatement; therefore, the falsification was judged as intentional.

3. Issues (data forgery, falsification) related to Supplementary Figure 5 and Figure 2F: The numbers of mice mentioned in the initially submitted version of the manuscript exhibited inconsistencies as a result of multiple instances of image duplication. Given the number of images duplicated at once, even between different experimental groups, this was considered to be the result of an intentional act.

4. Issues (data forgery, falsification) related to Supplementary Figure 10 and Figure 3E: This section revealed many instances of image duplication, causing inconsistencies between the original data and the numbers of mice mentioned in the initially submitted version of the manuscript. Moreover, this is not considered to be negligence. Given the number of overlapping images at a time, and also among different experimental groups, this was considered to be intentional.

5. Issues (data forgery, falsification) related to Supplementary Figure 15 and Figure 4E: The numbers of mice mentioned in the initially submitted version of the manuscript exhibited inconsistencies due to the duplication of multiple images. Given that so many instances of image duplication occurred simultaneously, these could not have been unintentional; therefore, data falsification was recognized as the result of a deliberate act.

Original article: Oncotarget. 2017; 8:65534–65547. 65534-65547. https://doi.org/10.18632/oncotarget.18032

